# Health Risk Assessments and Microbial Community Analyses of Groundwater from a Heavy Metal-Contaminated Site in Hezhou City, Southwest China

**DOI:** 10.3390/ijerph20010604

**Published:** 2022-12-29

**Authors:** Mingjie Xu, Kuankuan Zhang, Yiduo Wang, Bin Zhang, Kang Mao, Hua Zhang

**Affiliations:** 1 School of Architecture and Civil Engineering of Xihua University, Chengdu 610039, China; 2 State Key Laboratory of Environmental Geochemistry, Institute of Geochemistry, Chinese Academy of Sciences, Guiyang 550081, China; 3 College of Eco-Environment Engineering, Guizhou Minzu University, Guiyang 550025, China; 4 School of food and biotechnology of Xihua University, Chengdu 610039, China

**Keywords:** groundwater, heavy metals, health risks, microbial communities

## Abstract

In industrial site groundwater, heavy metal pollution is relatively common, causing great harm to the surrounding environment and human health. To explore the relationships between the heavy metal concentration, health risks and microbial community distribution, the groundwater from a polluted site at an abandoned processing plant in Hezhou City, China, is taken as the research object. A health risk assessment model recommended by the United States Environmental Protection Agency (US EPA) is used for the evaluation, and high-throughput sequencing technology is used to analyze the characteristics of the microbial community in the groundwater. The results show that the heavy metal pollution levels of five monitoring wells are different. The monitoring well labelled HLJ2 is polluted by Cu, Mn, Ni and Cd, and the other four monitoring wells are polluted by As and Cd to varying degrees. The carcinogenic risk values of heavy metals in the groundwater environments of the five monitoring wells are all greater than the acceptable range, and only the noncarcinogenic risk value of the HLJ2 monitoring well exceeds 1, which greatly impacts health. The risks posed by the contaminants in the site groundwater through the ingestion route of drinking water are greater than those caused by the ingestion route of skin contact. The groundwater environments of the five monitoring wells contain Proteobacteria and Patescibacteria, indicating that these two bacteria have certain tolerances to heavy metal pollution. The microbial community composition varies between the monitoring wells, suggesting that different concentrations and types of heavy metal contamination promote different types of bacterial growth. Studies have shown that Proteobacteria have many heavy metal resistance genes, improving their tolerance in heavy metal-polluted environments; additionally, Proteobacteria can transport heavy metals, which is conducive to the restoration of polluted sites.

## 1. Introduction

Groundwater accounts for approximately 30% of the total global freshwater resources, and the amount of groundwater is approximately 100 times greater than the total surface water resources [1]. Furthermore, groundwater constitutes nearly half of the world's drinking water [2]. In many areas in China, groundwater is the main source of drinking water, accounting for 17.5% of the total water supply, even nationally [3]. However, with the rapid development of machinery manufacturing, oil refinery, metal processing, and steel and automobile industries, some unreasonable behaviors, such as overexploitation, smelting and random industrial wastewater discharge, have seriously polluted groundwater [4,5,6]. According to the China Ecological Environment Status Report in 2020 issued by the Chinese government, among the 10,242 shallow groundwater environments monitored by relevant departments, 33.7 and 43.6% have poor and extremely poor water qualities, respectively [7]. With the improvement in the awareness of ecological environment protection strategies in China, an increasing number of high-pollution enterprises, such as mineral processing plants, have closed or relocated, leaving many polluted sites and posing a large threat to environmental safety and public health. Heavy metals have strong mobility, accumulation and toxicity characteristics, and they are difficult to degrade in groundwater. Heavy metals enter human bodies through the food chain, breath, diet and skin contact, which seriously threatens human health [8]. For example, excessive arsenic intake can damage the human immune system, leading to cancer of the kidneys, lungs, liver, skin and bladder [9]. Heavy metal contaminated water can cause long- and short-term effects, as well as diseases such as cirrhosis, renal failure, acute or chronic nervous system injury, cardiovascular disease, reproductive abnormality and cancer [10]. Therefore, a health risk assessment of such contaminated sites is needed.

Groundwater microorganisms are important parts of the subsurface biogeochemical cycle. Groundwater ecosystems serve as vast and complex habitats for different microbial communities, in which 6 to 40% of the prokaryotic microorganisms on earth inhabit [11]. Microorganisms can be used as response factors of groundwater pollution changes and reveal information about groundwater pollution [12]. In addition, microorganisms have certain resistance and detoxification characteristics to heavy metals, which can adsorb and transform heavy metals. Specific microbial communities control health risks by utilizing certain heavy metals as nutrients and converting them into low-toxicity and nontoxic components [13]. However, when the groundwater environment is seriously polluted, it also affects the microbial community in the groundwater. In recent years, high-throughput sequencing techniques have been applied to study microbial communities in different environments [14,15]. Therefore, special attention has been given to the changes in microbial community structures and functions after heavy metal contamination [16,17]. For example, numerous studies have shown that the stress of heavy metal pollution on the microbial community not only reduces the biomass of the microbial community in the environment but also affects the structure and function of the microbial community in the environment [18,19,20]. Heavy metals have different effects on different kinds of microorganisms [21]. Long-term exposure to heavy metals can lead to the enrichment of tolerant microbial populations, such as Proteobacteria and Firmicutes [22], whereas populations sensitive to heavy metals, such as α- Proteobacteria, may decrease [23]. The effects of different types of heavy metals on microbial communities were also different. For example, the contribution of Cr to microbial α-diversity was much higher than that of Mn [24]. Therefore, heavy metals can affect the microbial communities involved in various biological earth cycle processes and ecosystem functions, thereby affecting their ability to degrade pollutants [25]. Through the risk assessment of the groundwater in the contaminated site, the types of pollutants and the degree of pollution in different places in the site are clear. At the same time, the assessment results explain the composition of the microbial community structure in the groundwater to explore the impact of environmental factors on microbial communities, which provides a theoretical basis for subsequent site remediation.

The main products of the factory in the research site are arsenic trioxide, metallic arsenic and ammonium molybdate; highly soluble heavy metals easily migrate and transform in soil, water and other media, causing a large area of soil and groundwater pollution and posing a huge security risk to the ecological environment and human health. At the same time, the area has less precipitation and strong evaporation. Groundwater is the main water source for the surrounding residents, and its water quality is of great significance to human health. There is a drinking water source protection area in the southeast of the site, and pollutants in the site may pollute the water source through surface runoff, groundwater recharge, etc. In addition, karst caves are abundant in karst areas, and the groundwater is very vulnerable to pollution; the polluted groundwater will reach other areas through developed caves. Therefore, it is particularly important to understand the groundwater pollution status and health risks of the site. In this study, the polluted site of an abandoned processing plant in Hezhou City, China, is selected as the research object, and the heavy metals Cr, As, Cd, Pb, Cu, Mn and Ni are selected as the pollutants of interest. Using the health risk assessment model recommended by the US EPA, the health risk assessment of heavy metals in groundwater was conducted, and the characteristics of the groundwater microbial community were analyzed. This study is expected to serve as a reference for the assessment of pollution levels, distribution characteristics and health risks of heavy metals in soil and groundwater at the site; additionally, this study can aid with evaluating the abilities of microorganisms to remediate sites.

## 2. Materials and Methods

### 2.1. Study Area

**Figure 1:**
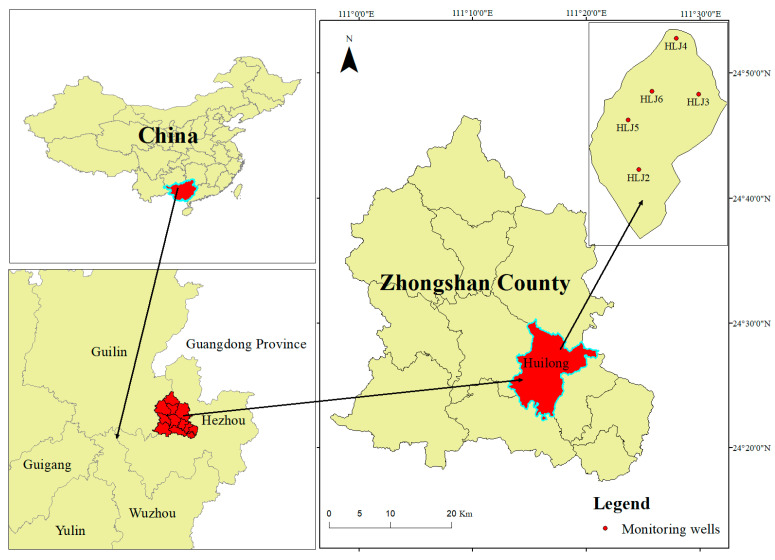
Location of the study area and monitoring well distribution.

Zhongshan County is located in the northeastern Guangxi Zhuang Autonomous Region, with geographic coordinates of 110°58′~111°31′ E, 24°17′~24°46′ N (Figure 1). The research site is located in Huilong Town, Zhongshan County, and it has a subtropical monsoon climate, with an average temperature of 19.7 °C and annual precipitation of 1576.7 mm, mainly concentrated from April to June. The maximum annual precipitation is 2371.4 mm, and the minimum precipitation is 1091 mm. According to the groundwater occurrence conditions, water-bearing media and hydraulic characteristics, the groundwater types in the study area are divided into two categories: loose rock pore water and carbonate rock fissures/karst cave water. The pore water levels of loose rocks are mainly distributed in low-lying regions in the survey area. The carbonate rock fissures and karst caves in the survey area are hosted in the limestone aquifer formation of the Upper Devonian Guilin Formation, which is controlled by the development degrees of karst caves, crevices and various pores. The groundwater in the area where the site is located is mainly stagnant water in the upper layer that occurs in the covering layers, such as miscellaneous fill and silty clay. There is no uniform water level. During the survey, the stable water levels of some boreholes were measured at burial depths of 2.18–6.8 m.

### 2.2. Sample Collection

According to the technical specifications for groundwater environmental monitoring (HJ/T164-2020) on the selection of sampling points, sampling methods and requirements, in addition to the site topography and groundwater flow direction, a total of 5 groundwater monitoring wells were set up (HLJ2, HLJ3, HLJ4, HLJ5, HLJ6; Figure 1). In April 2022, samples were collected from 5 groundwater sampling points, and 3 parallel samples were collected for each detection index at each sampling point for a total of 30 samples. Before collecting samples, the wells were cleaned. After well cleaning, the pH value, conductivity, temperature, oxidation reduction potential and dissolved oxygen of groundwater in each monitoring well were measured, and the readings had to be stable within ±10% before groundwater sample collection. The DZB-712 portable multiparameter analyzer (Shanghai Thunder Magnetic Instrument Co., Ltd., Shanghai, China) was used on site to measure and record the fundamental water quality indicators, such as pH, water temperature, dissolved oxygen and electrical conductivity, at each sampling point. The collection container was a 50 mL polyethylene centrifuge tube, which was rinsed three times before sampling with the water sample at the sampling point. The samples used for detecting heavy metals (Cr, Ni, Cu, As, Cd, Pb, Mn) were filtered through a 0.45-μm microporous membrane and then acidified to pH < 2 by adding premium pure HNO_3_. The samples used to detect anions (F^−^, SO_4_^2−^, Cl^−^, NO_3_^−^) were directly sealed after being filtered through a 0.45-μm microporous membrane. Microorganisms in 2 L groundwater samples were filtered with a 0.22-μm filter membrane until the surface of the filter membrane was covered with visible cover, and then the filter membrane was placed in a 15 mL polyethylene test tube for preservation. All the above samples were stored in a 4 °C refrigerator protected from light and removed for laboratory analysis.

### 2.3. Analytical Methods

An inductively coupled plasma mass spectrometer (Agilent ICP-MS 8900, Agilent Technology Co. Ltd., Santa Clara, CA, USA) was used to detect the concentrations of heavy metals in the samples. The standard curve was prepared using the standard materials provided by the National Standards Centre of China. The average recovery rate of internal standards for heavy metals is between 95 and 125%, and the relative standard deviation (RSD) values of heavy metals were less than 5%. The chemicals used in the experiment were all high-grade, pure samples. A Dionex ICS-90 ion chromatograph (Yubo Industry Co., Ltd., Shanghai, China) was used to detect the concentrations of anions in the sample. An AS40 type autosampler was used to introduce the sample with the following detection range: digital mode 0~1000 μs/cm (resolution 0.2 nS/cm), conductivity cell volume <1 μL and electron drift <5 nS/h.

The total DNA was extracted using a power soil DNA isolation kit (MOBIO Laboratories, Carlsbad, CA, USA), and the integrity and purity of the DNA were detected using 1% agarose gel electrophoresis. Using primers 338F (5′-ACTCCTACGGGAGGCAGCA-3′) and 806R (5′-GGACTACHVGGGTTWTCTAAT-3′), the variable regions of 16S rDNA gene V3~V4 were amplified using polymerase chain reactions (PCRs). The constructed amplicon library was sequenced using an Illumina Nova 6000 platform (Guangdong Magigene Biotechnology Co., Ltd., Guangzhou, China), and usearch10 software (Guangdong Magigene Biotechnology Co., Ltd., Guangzhou, China) was used to perform operational taxonomic unit (out) clustering for the sequences according to 97% similarity and to propose chimaeras. The representative sequences of each OTU were aligned with the SILVA (16S) database using usearch sintax to obtain species annotation information.

### 2.4. Health Risk Assessment

Health risk assessments are a method for quantitatively describing the risk of pollution to human health by linking environmental pollution to human health while using risk as an evaluation index [26]. Pollutants in groundwater enter the human body through two exposure routes: drinking water ingestion and dermal contact [27]. According to the land use released by the relevant departments of Zhongshan County, Hezhou City, the research area is planned to be a park green space in the future. In accordance with the technical guidelines for soil pollution risk assessment of construction land (HJ 25.3-2019), the risk assessment is conducted for the first type of land represented by the green space. Under the first type of land use, both children and adults may be exposed long-term to contaminated land, causing health hazards. In terms of carcinogenic effects, the lifetime exposure hazards of the population are considered, and the lifetime carcinogenic risks of pollutants are generally assessed based on exposure in childhood and adulthood; as children are lighter in weight and receive greater exposure than adults, noncarcinogenic effects are generally determined based on childhood exposure. The noncarcinogenic hazard effects of pollutants are assessed. As the health risk assessment model recommended by the US EPA is widely used and suitable for this research site, this model was selected in this work. In accordance with the World Health Organization (WHO), the International Agency for Research on Cancer (IARC), human epidemiological research and other data were used to classify pollutants and perform carcinogenic and noncarcinogenic risk assessments for the heavy metals studied in this paper [28,29]. However, the calculation parameters of the same element under different exposure routes are different, and the calculations of carcinogenic and noncarcinogenic risks under the same exposure routes are different. The exposure parameters in this study are shown in Table S1, and the skin permeability coefficient, carcinogenic slope factor and exposure reference dose are shown in Table S2 [30,31,32,33,34,35].

#### 2.4.1. Determination of Exposure

The formula for calculating the average daily exposure dose (ADD_oral_) of noncarcinogenic effects under the drinking water ingestion route of a single pollutant is as follows:

(1) $${\mathrm{ADD}}_{\mathrm{oral}}=\frac{c\times {\mathrm{IR}}_{c}\times {\mathrm{EF}}_{c}\times {\mathrm{ED}}_{c}}{{\mathrm{BW}}_{c}\times {\mathrm{AT}}_{\mathrm{nc}}}$$

where c (mg/L) is the average concentration of pollutants in groundwater; IR_c_ (L/d) is the average daily water ingestion of children; EF_c_ (d/a) is the average exposure frequency of children; ED_c_ (a) is the average exposure period of children; BW_c_ (kg) is the average body weight of children; and AT_nc_ (d) is the average exposure time to noncarcinogenic effects.

The formula for calculating the average daily exposure dose (LADD_oral_) of carcinogenic effects under the drinking water ingestion route of a single pollutant is as follows:

(2) $${\mathrm{LADD}}_{\mathrm{oral}}=\frac{c\times {\mathrm{IR}}_{a}\times {\mathrm{EF}}_{a}\times {\mathrm{ED}}_{a}}{{\mathrm{BW}}_{a}\times {\mathrm{AT}}_{\mathrm{ca}}}+\frac{c\times {\mathrm{IR}}_{c}\times {\mathrm{EF}}_{c}\times {\mathrm{ED}}_{c}}{{\mathrm{BW}}_{c}\times {\mathrm{AT}}_{\mathrm{ca}}}$$

where IR_a_ (L/d) is the average daily water ingestion of adults; EF_a_ (d/a) is the average exposure frequency of adults; ED_a_ (a) is the average exposure period of adults; BW_a_ (kg) is the average body weight of adults; and AT_ca_ (d) is the average exposure time to carcinogenic effects.

The formula for calculating the average daily exposure dose (ADD_dermal_) of noncarcinogenic effects under the dermal contact route of a single pollutant is as follows:

(3) $${\mathrm{ADD}}_{\mathrm{dermal}}=\frac{c\times {\mathrm{SA}}_{c}\times \mathrm{PC}\times \mathrm{CF}\times {\mathrm{EF}}_{c}\times {\mathrm{ET}}_{c}\times {\mathrm{ED}}_{c}}{{\mathrm{BW}}_{c}\times {\mathrm{AT}}_{\mathrm{nc}}}$$

where SA_c_ (cm^2^) is the exposed surface area of children's skin; PC (cm/h) is the skin permeability coefficient; CF (L/cm^3^) is the volume conversion factor; and ET_c_ (h/d) is the daily exposure time of children's skin to pollutants.

The formula for calculating the average daily exposure dose (LADD_dermal_) of carcinogenic effects under the dermal contact route of a single pollutant is as follows:

(4) $${\mathrm{LADD}}_{\mathrm{dermal}}=\frac{c\times {\mathrm{SA}}_{a}\times \mathrm{PC}\times \mathrm{CF}\times {\mathrm{EF}}_{a}\times {\mathrm{ET}}_{a}\times {\mathrm{ED}}_{a}}{{\mathrm{BW}}_{a}\times {\mathrm{AT}}_{\mathrm{ca}}}+\frac{c\times {\mathrm{SA}}_{c}\times \mathrm{PC}\times \mathrm{CF}\times {\mathrm{EF}}_{c}\times {\mathrm{ET}}_{c}\times {\mathrm{ED}}_{c}}{{\mathrm{BW}}_{c}\times {\mathrm{AT}}_{\mathrm{ca}}}$$

where SA_a_ (cm^2^) is the exposed surface area of adult skin, and ET_a_ (h/d) is the daily exposure time of adult skin to pollutants.

#### 2.4.2. Characterization of Health Risks

The formula for calculating the carcinogenic risk value (CR_oral_) of a single pollutant through the drinking water ingestion route is as follows:

(5) $${\mathrm{CR}}_{\mathrm{oral}}={\mathrm{LADD}}_{\mathrm{oral}}\times {\mathrm{SF}}_{o}$$

where SF_o_ ((mg/kg/d)^−1^) is the carcinogenic slope factor of the drinking water ingestion route.

The formula for calculating the noncarcinogenic risk value (HQ_oral_) of a single pollutant through the drinking water ingestion route is as follows:

(6) $${\mathrm{HQ}}_{\mathrm{oral}}=\frac{{\mathrm{ADD}}_{\mathrm{oral}}}{{\mathrm{RfD}}_{o}}$$

where RfD_o_ (mg·(kg·d)^−1^) is the noncarcinogenic average daily exposure reference dose of the drinking water ingestion route.

The formula for calculating the carcinogenic risk value (CR_dermal_) of a single pollutant through the dermal contact route is as follows:

(7) $${\mathrm{CR}}_{\mathrm{dermal}}={\mathrm{LADD}}_{\mathrm{dermal}}\times {\mathrm{SF}}_{d}$$

where SF_d_ ((mg/kg/d)^−1^) is the carcinogenic slope factor of the dermal contact route.

The formula for calculating the noncarcinogenic risk value (HQ_oral_) of a single pollutant through the dermal contact route is as follows:

(8) $${\mathrm{HQ}}_{\mathrm{dermal}}=\frac{{\mathrm{ADD}}_{\mathrm{dermal}}}{{\mathrm{RfD}}_{d}}$$

where RfD_d_ (mg·(kg·d)^−1^) is the noncarcinogenic average daily exposure reference dose for the dermal contact route.

Assume that the health risk effects of each pollutant on the human body are additive rather than antagonistic or synergistic [36]. Under a certain exposure route, the calculation formulas of the total carcinogenic risks (TCR) of pollutants in groundwater and the total noncarcinogenic hazard indices (HIs) of pollutants in groundwater are as follows:

(9) $$\mathrm{TCR}=\sum {\mathrm{CR}}_{\mathrm{oral}/\mathrm{dermal}}\;\mathrm{HI}=\sum {\mathrm{HQ}}_{\mathrm{oral}/\mathrm{dermal}}$$

If HI > 1, the noncarcinogenic contaminant has a negative impact on human health; if HI < 1, the noncarcinogenic risks of exposed individuals are within acceptable limits.

## 3. Results and Discussions

### 3.1. Distribution Characteristics of Heavy Metals in Groundwater

The concentrations of heavy metals in the groundwater of each monitoring well are shown in Table 1. The concentrations of heavy metals in the five monitoring wells are different, and the concentration of As is much higher than the concentrations of other heavy metals. The concentrations of Mn, Cd, Ni and Cu in the monitoring well HLJ2 are the highest among the five monitoring wells, and the concentration of As in the monitoring well HLJ6 is the highest. The Cr and Pb concentrations in each monitoring well are lower than the class III concentration threshold of the Chinese Groundwater Quality Standard (GB/T14848-2017). The concentrations of Mn, Cd, Ni and Cu in the HLJ2 monitoring well are 221.3, 81.67, 11.18 and 1.05 times the standard values, respectively. The concentrations of As and Cd in the monitoring wells HLJ3–HLJ6 are higher than the standard limit. The arsenic pollution at the whole site is relatively serious, and the average concentration exceeds 5300 times the standard value. This result is related to the nature of the research site. Before the site was abandoned, it was an arsenic processing plant, and the main product was arsenic trioxide. Due to the immature treatment technology at the time, the production wastewater was directly discharged or stayed in place, resulting in groundwater pollution at the site.

### 3.2. Correlation Analysis

Using SPSS software, the Spearman correlation coefficient was used to analyze the correlations of various pollutant concentrations at the site. The Spearman correlations are shown in Table 2 and Table 3. The correlation analyses between heavy metals show that there are significant positive correlations between Cr, Cu, Pb, Mn and Cd, indicating that Cr, Cu, Pb, Mn and Cd undergo similar environmental geochemical effects [37]. The correlation coefficient between Cu and Pb is the highest at 0.968, indicating that Cu and Pb are closely related and greatly influence each other. There are significant positive correlations between Mn and Cr, Mn and Cd, and Mn and Ni, indicating that Mn, Cr, Cd and Ni have the same source. There are significant negative correlations between As and Ni, and As and Mn, and there are no significant correlations with other pollutants, indicating that As differs from the other pollutants.

According to the correlation coefficient between heavy metals and the other components (Table 3), the pH level of the study site is significantly negatively correlated with Cd, Cu and Pb; As is significantly negatively correlated with F^−^, Cl^−^, NO_3_^−^, K^+^ and Na^+^, whereas there are significant positive correlations between Mn and F^−^, Cl^−^ and K^+^. There is a positive correlation between pH and As. Arsenic exists in groundwater in an uncharged or negatively charged form and is easily adsorbed by positively charged substances in water. When the pH increases, the colloid or clay minerals have more negative charges, reducing their adsorption capacity for As and resulting in an increase in the As concentration in groundwater [38]. There is a significant negative correlation between As and NO_3_^-^. When the concentration of NO_3_^−^ in water is high, the iron and manganese oxides are more stable. Additionally, the reduction reactions of arsenic-containing iron and manganese oxides do not occur to release arsenic [39].

### 3.3. Human Health Risk Assessment

According to the health risk assessment models and parameters, the carcinogenic and noncarcinogenic risk values of pollutants in the groundwater of the research site through drinking water ingestion and dermal contact routes were calculated, and the results are shown in Table 4 and Table 5. The carcinogenic risk of groundwater pollutants at the research site is relatively high; only the carcinogenic risk value of the population exposed to the dermal contact route from the HLJ2 monitoring well is under the maximum acceptable risk level recommended by the International Association for Research on Cancer (5.0 × 10^−5^). The order of the total carcinogenic risk of the five monitoring wells is HLJ6 > HLJ5 > HLJ4 > HLJ3 > HLJ2. The order of carcinogenic risk caused by the three elements is As > Cd > Cr. The carcinogenic risk values of As and Cd are close to or higher than the maximum acceptable level, whereas Cr is lower than the maximum acceptable level. Most of the noncarcinogenic risks of the groundwater pollutants in the study site are less than 1, and only the noncarcinogenic risks of the HLJ2 monitoring well exposed through drinking water ingestion are at a higher risk (HI > 1). The total noncarcinogenic risk of the five monitoring wells is as follows: HLJ2 > HLJ5 > HLJ3 > HLJ6 > HLJ4. The order of the noncarcinogenic risks caused by the four elements is Mn > Cu > Ni > Pb, in which the noncarcinogenic risk value of Cu and Mn caused by the skin contact route is greater than 1, indicating that people exposed to the groundwater of the site cause noncarcinogenic risks. However, the noncarcinogenic risks caused by Ni and Pb through drinking water ingestion and dermal contact routes are all within acceptable ranges. The total carcinogenic risk under the two exposure routes of drinking water ingestion and dermal contact range from 3.56 × 10^−2^ to 2.43. The health risk is relatively high and has a serious impact on the health of the population. The noncarcinogenic risks under the two exposure routes are mostly within the acceptable range, indicating that the health risks caused by groundwater pollutants in the study site are mainly from carcinogenic elements, especially As; the total carcinogenic risk caused by the heavy metals exceed the acceptable value by 4~5 orders of magnitude. In addition, the carcinogenic and noncarcinogenic risks of contaminants in groundwater exposed through drinking ingestion are higher than those through dermal exposure, indicating that the drinking ingestion route is the main exposure route of contaminants in groundwater [40,41].

### 3.4. Microbial Community Analysis in Groundwater

#### 3.4.1. Microbial Community Composition Based on Phylum and Genus Levels

**Figure 2:**
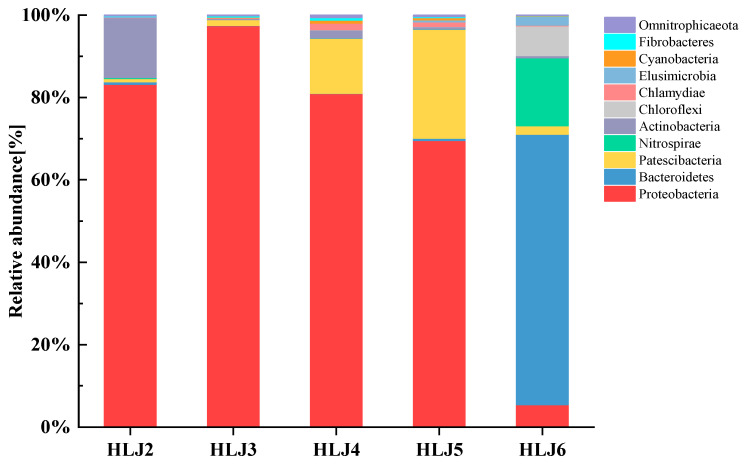
Relative abundance levels of the microbial communities at the phylum level in each monitoring well.

Changes in microbial community structures can reflect the distribution of microorganisms [42]. Based on the results of the microbial high-throughput sequencing of 16S rDNA, the relationship between groundwater microbial community composition and heavy metal health risks was explored. The ten phyla and genera with the highest relative abundance were selected for research. The relative abundance of the microbial community in each monitoring well of the site at the phylum level is shown in Figure 2, which shows that the relative abundance levels of bacterial phyla in the HLJ2 monitoring well are Proteobacteria, Actinobacteria, Patescibacteria, Bacteroidetes and Elusimicrobia, listed in order from high to low. The HLJ3 monitoring well is followed by Proteobacteria, Patescibacteria, Fibrobacteres, Actinobacteria and Omnitrophicaeota; the HLJ4 monitoring well is followed by Proteobacteria, Patescibacteria, Actinobacteria, Chlamydiae and Cyanobacteria; the HLJ5 monitoring well is followed by Proteobacteria, Patescibacteria, Chlamydiae, Cyanobacteria and Bacteroidetes; and the HLJ6 monitoring well is followed by Bacteroidetes, Nitrospirae, Chloroflexi, Elusimicrobia and Patescibacteria. The five monitoring wells all contain Proteobacteria, and Proteobacteria account for the largest proportion of the phylum in the HLJ2–HLJ5 monitoring wells, with relative abundance levels ranging from 69.6 to 97.5%. Proteobacteria are found to dominate other types of polluted environments, such as heavy metal-contaminated soil [43], sewer pipes [44] and sewage sludge [45]. Proteobacteria exhibit high tolerance levels in heavy metal-contaminated environments due to their remarkable metabolic diversity, especially considering the various physiologies of respiratory mechanisms, including obligate aerobes and facultative and obligate anaerobic bacteria [46]. In addition, Proteobacteria are rich in heavy metal resistance genes, especially Gammaproteobacteria. With the help of heavy metal resistance genes, Proteobacteria can transport heavy metal ions and use enzymes to detoxify them, allowing them to act as microbes that remediate certain heavy metal-contaminated sites [47]. The relative abundance of Bacteroidetes in the HLJ6 monitoring well accounts for the largest proportion. Studies have shown that Bacteroidetes are fermentative and sulphate-reducing microorganisms that possess many heavy metal resistance genes; they are the main bacterial group in heavy metal-polluted environments [48]. The relative abundance levels of the other species of bacteria in the five monitoring wells are lower, which may be related to the lower tolerances of these species to heavy metals.

**Figure 3:**
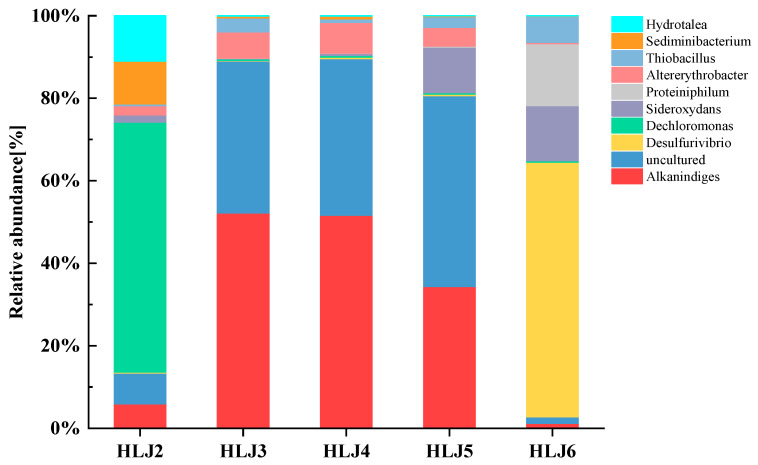
Relative abundance levels of microbial communities at the genus level in monitoring wells.

The relative abundance levels of microbial communities at the genus level in the groundwater of the five monitoring wells are shown in Figure 3. *Dechloromonas* (60.59%) dominates the HLJ2 monitoring well, *Alkanindiges* (52.19%, 51.62%) dominates the HLJ3 and HLJ4 monitoring wells, *Uncultured* (46.27%) dominates the HLJ5 monitoring well, and *Desulfurivibrio* (61.72%) dominates the HLJ6 monitoring well. *Desulfurivibrio* is a sulphate-reducing bacterium that produces sulphides that play multiple roles in arsenic enrichment in groundwater, including the inorganic reduction in arsenic-containing oxidized minerals to release adsorbed arsenic [49] and the formation of dissolved arsenic sulphide or arsenic acid reduction to arsenite to improve arsenic mobility in groundwater [50,51], which may be the reason for the high As concentration in the HLJ6 monitoring well. There are some differences between the five monitoring wells at the genus level. *Alkanindiges* and *Uncultured* have high abundance levels in the HLJ3, HLJ4 and HLJ5 monitoring wells, whereas their relative abundance levels are low in the HLJ6 monitoring well. A bacteria with a relative abundance level over 5% is defined as the dominant genus [52]. *Dechloromonas*, *Sediminibacterium* and *Hydrotalea* are the dominant genera in the HLJ2 monitoring well, whereas the relative abundance levels in the other four monitoring wells are all under 1%. The bacterial heterogeneity suggests that different levels of heavy metal pollution lead to different microbial community structures [52]. The succession of the microbial community is the result of the selection of the surrounding environment, but the specific succession law of the microbial community at this site is still unclear. The specific succession law of the groundwater microbial community needs to be further studied.

#### 3.4.2. Microbial Community Diversity

**Figure 4:**
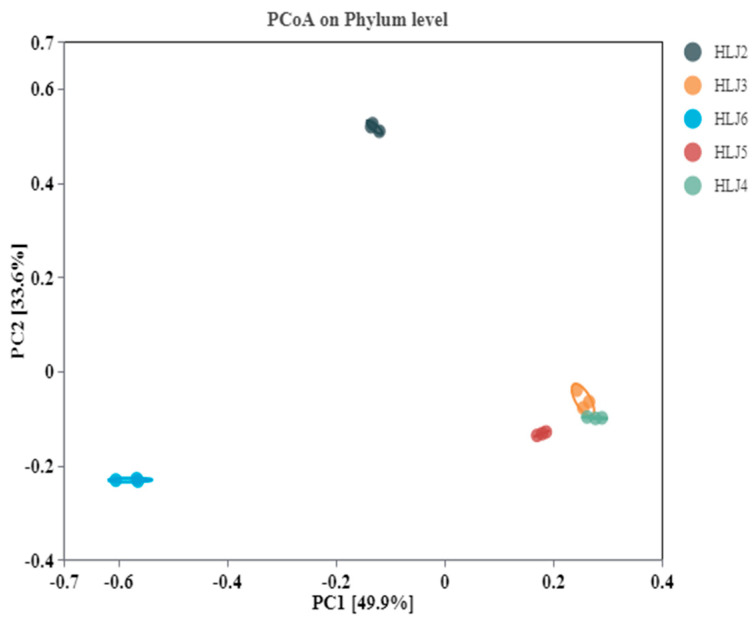
Principal coordinate analyses (PCoA) of bacterial communities.

The alpha diversity indices of microorganisms in different monitoring wells are shown in Table 6. Chao1 is an index reflecting the abundance of the community, and the larger the value is, the greater the total number of species. Shannon is an index reflecting the community diversity, and the larger the value is, the greater the diversity of species. Simpson is an index for evaluating the uniformity of the species. When the Simpson index is close to 1, the distribution of microbial communities is more uniform [53]. The Chao1 index shows (Table 6) that the community abundance of the HLJ5 monitoring well is higher than that of the other monitoring wells; the community abundance of the HLJ2 monitoring well is the smallest, but its uniformity is the highest. The HLJ6 monitoring well has the highest Shannon index, indicating the highest community diversity. Studies have shown that bacterial communities are sensitive to changes in environmental conditions, especially in the presence of heavy metals [54]. Compared to the microbial diversity levels in other heavy metal-contaminated areas [55,56], the microbial richness and diversity of the research site are low, which is attributed to the serious pollution of the site. To better assess the changes in the bacterial communities in the groundwater of the five monitoring wells of the contaminated site, we performed principal coordinate analysis (PCoA) at the phylum level (Figure 4) to further determine the similarity and dissimilarity levels of the communities. The results show that the two main selected axes—PC1 and PC2—together explain 83.5% of the total variation in the bacterial community structure. The HLJ2 and HLJ6 monitoring wells are the farthest along the PC2 axis, indicating that the bacterial community structure between them is quite different. In the direction of the PC1 axis, the interval between the HLJ6 and HLJ4 monitoring wells is the largest, which indicates that the bacterial community structure between the two is quite different, and the PC1 axis is the main factor contributing to the difference. The three monitoring wells—HLJ3, HLJ4 and HLJ5—have small intervals in the directions of the two main coordinate axes, indicating that the bacterial community structures of the three monitoring wells have few differences.

#### 3.4.3. Correlation between Bacterial Community and Environmental Parameters

**Figure 5:**
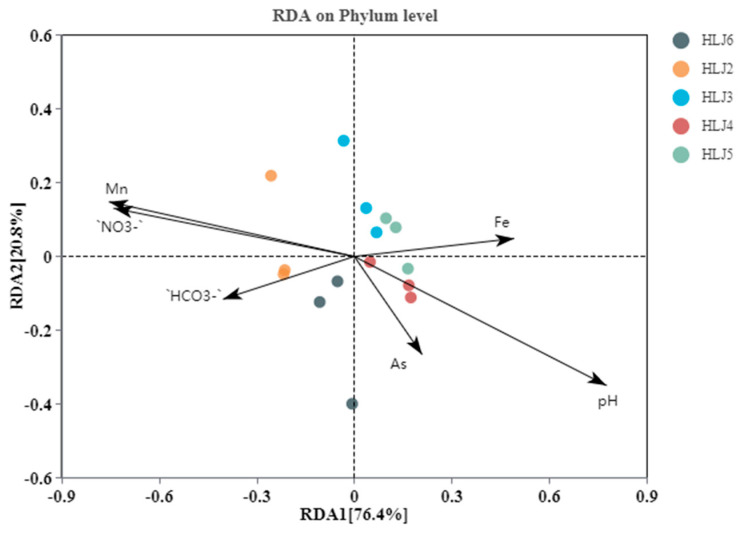
RDAs between phylum-level microbial community structure and environmental factors.

At the phylum level, the relative abundance levels of microorganisms and the main environmental parameters were analyzed using redundancy analysis (RDA) to explore the influences of environmental factors on the microbial community structures in groundwater. From Figure 5, the first main selection (RDA1) explains 76.4% of the environmental factors on bacterial community diversity, and the second main selection axis (RDA2) explains 20.8% of the environmental factors on bacterial community diversity. The environmental factors that greatly influence the microbial community structure in the HLJ6 monitoring well are Mn and NO_3_^−^, but pH has little effect on them. The concentrations of Mn and NO_3_^−^ in groundwater are negatively correlated with the microbial community composition in the HLJ4 monitoring well but positively correlated with the other four monitoring wells. Studies have shown that high concentrations of NO_3_^−^ make groundwater an anoxic environment, which is not conducive to the reduction and dissolution of As-containing ferric hydroxide [57], resulting in lower As concentrations in the groundwater of the HLJ4 monitoring well than in the other monitoring wells. pH levels have greater impacts on the microbial community structures of the HLJ3, HLJ4 and HLJ5 monitoring wells, and Fe, HCO_3_^−^ and As have weaker effects on the microbial community structure of the five monitoring wells than Mn and NO_3_^-^. In addition, As is positively correlated with pH and Fe and negatively correlated with other environmental factors.

## 4. Conclusions

The groundwater of the current site is polluted by different heavy metals. The main pollutants in the HLJ2 monitoring well are Cu, Mn, Ni and Cd, and the main pollutants in the HLJ3–HLJ6 monitoring wells are Cd and As. The average As concentration of the entire site is over 5300 times the standard limit of class III groundwater. As only has a significant correlation with Ni and Mn and has significant correlation with other pollutants. As is positively correlated with pH and SO_4_^2−^ and negatively correlated with other anions and cations. The carcinogenic risk of groundwater at the current site is high, and the total carcinogenic risk value ranges from 3.56 × 10^−2^ to 2.43. The total noncarcinogenic risk value of each monitoring well is only greater than 1 in HLJ2, and drinking ingestion is the main route of exposure. The compositions of the microbial communities in the groundwater environments of the five monitoring wells are different. The microbial community structures of the HLJ3, HLJ4 and HLJ5 monitoring wells are similar, whereas the microbial community structure of the HLJ2 monitoring well is slightly different, and the HLJ6 monitoring well is obviously different from the others. Significantly, different levels and different heavy metal pollutions create the unique microbial community structure of each monitoring well. The five monitoring wells contain different abundance levels of Proteobacteria, which have good resistance to heavy metals and are expected to serve as strategies for remediating heavy metal-contaminated sites. In addition, Mn and NO_3_^−^ have the greatest impact on microbial community structures, and other environmental factors are weaker than the former two. To sum up, from the perspective of environmental safety, it is necessary to control the As pollutants to prevent their migration from polluting the surrounding groundwater and soil. Each monitoring well is polluted to different degrees. It is recommended to select appropriate remediation technologies to conduct the cost-effective restoration of the site according to the health risks of the different monitoring wells. The five monitoring wells all contain Proteobacteria, which are relatively abundant in the HLJ3 monitoring well, whereas the monitoring well is relatively low in pollution, indicating that it has a good ability to degrade pollutants. The degradation mechanism of heavy metals by different Proteobacteria species is the focus of future research. However, microbial abundance does not reflect changes in the population structure. Microbial community structures cannot accurately reveal the performance or function of microorganisms. To accurately reflect the quality of groundwater, it is necessary to strengthen the diagnostic research of functional microorganisms on the quality of groundwater contaminated by heavy metals. In addition, microorganisms are extremely sensitive to environmental changes and are easily affected by environmental factors such as temperature and organic matter. Therefore, it is necessary to strengthen the research on the relationship between the physical and chemical factors of groundwater and microorganisms. The concentration of heavy metals in different seasons will also show different changes, which will lead to changes in the microbial community. In the future, changes in heavy metal concentrations and microbial communities in different seasons should be studied to further elaborate the relationship between microbial communities and heavy metals.

## Figures and Tables

**Table 1:** Distribution of heavy metal concentrations in groundwater (μg/L).

Heavy Metals	Monitoring Well Number					Standard *
	HLJ2	HLJ3	HLJ4	HLJ5	HLJ6	
Mn	22,130 ± 138.41	370 ± 1.34	250 ± 0.30	330 ± 1.78	250 ± 1.93	100
Cr	0.38 ± 0.15	0.28 ± 0.16	0.09 ± 0.05	0.19 ± 0.05	0.25 ± 0.10	5
As	16.13 ± 1.14	19,407.34 ± 124.03	31,733.05 ± 458.42	106,242.67 ± 1482.80	111,640.67 ± 1286.29	10
Cd	408.33 ± 11.76	19.85 ± 1.26	14.31 ± 0.44	23.35 ± 0.54	18.58 ± 0.82	5
Pb	8.95 ± 0.24	0.26 ± 0.04	0.53 ± 0.22	2.31 ± 0.10	1 ± 0.67	10
Cu	1054.92 ± 39.75	0.28 ± 0.22	2.6 ± 1.05	8.43 ± 1.57	4.16 ± 0.52	1000
Ni	223.53 ± 7.86	4.46 ± 0.11	3.82 ± 0.27	1.8 ± 0.01	1.66 ± 0.15	20

* Class III concentration threshold of the Chinese Groundwater Quality Standard (GB/T14848-2017).

**Table 2:** Spearman correlation coefficients between heavy metals in groundwater.

Heavy Metals	Cr	As	Cd	Ni	Cu	Pb	Mn
Cr	1.000	−0.182	0.621 *	0.282	0.318	0.325	0.564 *
As		1.000	−0.414	−0.950 **	−0.111	−0.157	−0.671 **
Cd			1.000	0.443	0.746^**^	0.746 **	0.854 **
Ni				1.000	0.132	0.182	0.682 **
Cu					1.000	0.968 **	0.407
Pb						1.000	0.439
Mn							1.000

Note: * Correlation is significant at the 0.05 level (2-tailed); ** Correlation is significant at the 0.01 level (2-tailed).

**Table 3:** Spearman correlation coefficients between groundwater metal elements and other components.

	Cr	As	Cd	Ni	Cu	Pb	Mn
pH	−0.286	0.097	−0.699 *	−0.122	−0.954 **	−0.894 **	−0.432
F^-^	0.418	−0.589 *	0.596 *	0.554 *	−0.021	0.043	0.864 **
Cl^-^	0.493	−0.568 *	0.607 *	0.586 *	−0.029	0.036	0.854 **
NO_3_^-^	0.054	−0.871 **	0.239	0.861 **	0.207	0.250	0.371
SO_4_^2-^	0.257	0.182	0.093	−0.182	−0.411	−0.375	0.307
K^+^	0.461	−0.521 *	0.375	0.479	−0.143	−0.082	0.739 **
Na^+^	0.229	−0.757 **	0.021	0.768 **	−0.375	−0.300	0.479

Note: * Correlation is significant at the 0.05 level (2-tailed); ** Correlation is significant at the 0.01 level (2-tailed).

**Table 4:** Results of the carcinogenic risk assessments by drinking water ingestion and the dermal contact route.

Route of Exposure	Heavy Metals	Monitoring Well Number				
		HLJ2	HLJ3	HLJ4	HLJ5	HLJ6
Drinking water ingestion	Cr	2.67 × 10^−6^	1.96 × 10^−6^	6.54 × 10^−7^	1.34 × 10^−6^	1.80 × 10^−6^
	As	3.42 × 10^−4^	4.12 × 10^−1^	6.74 × 10^−1^	2.26	2.37
	Cd	3.53 × 10^−2^	1.71 × 10^−3^	1.24 × 10^−3^	2.02 × 10^−3^	1.60 × 10^−3^
	TCR	3.56 × 10^−2^	4.14 × 10^−1^	6.75 × 10^−1^	2.26	2.37
Dermal contact	Cr	1.29 × 10^−6^	9.47 × 10^−7^	3.17 × 10^−7^	6.48 × 10^−7^	8.70 × 10^−7^
	As	9.09 × 10^−6^	1.09 × 10^−2^	1.79 × 10^−2^	5.99 × 10^−2^	6.29 × 10^−2^
	Cd	2.66 × 10^−5^	1.29 × 10^−6^	9.31 × 10^−7^	1.52 × 10^−6^	1.21 × 10^−6^
	TCR	3.70 × 10^−5^	1.09 × 10^−2^	1.79 × 10^−2^	5.99 × 10^−2^	6.29 × 10^−2^

**Table 5:** Noncarcinogenic risk assessment results of drinking water ingestion and the dermal contact route.

Route of Exposure	Heavy Metals	Monitoring Well Number				
		HLJ2	HLJ 3	HLJ 4	HLJ 5	HLJ 6
Drinking water ingestion	Ni	5.13 × 10^−1^	1.02 × 10^−2^	8.76 × 10^−3^	4.13 × 10^−3^	3.82 × 10^−3^
	Cu	1.21	3.22 × 10^−4^	2.98 × 10^−3^	9.68 × 10^−3^	4.77 × 10^−3^
	Pb	2.94 × 10^−1^	8.66 × 10^−3^	1.73 × 10^−2^	7.56 × 10^−2^	3.29 × 10^−2^
	Mn	2.21 × 10	3.70 × 10^−1^	2.48 × 10^−1^	3.31 × 10^−1^	2.53 × 10^−1^
	HI	2.41 × 10	3.89 × 10^−1^	2.77 × 10^−1^	4.20 × 10^−1^	2.94 × 10^−1^
Dermal contact	Ni	1.10 × 10^−3^	2.20 × 10^−5^	1.88 × 10^−5^	8.86 × 10^−6^	8.18 × 10^−6^
	Cu	2.34 × 10^−2^	6.22 × 10^−6^	5.76 × 10^−5^	1.87 × 10^−4^	9.21 × 10^−5^
	Pb	6.80 × 10^−6^	2.01 × 10^−7^	4.01 × 10^−7^	1.75 × 10^−6^	7.63 × 10^−7^
	Mn	3.27 × 10^−1^	5.48 × 10^−3^	3.67 × 10^−3^	4.90 × 10^−3^	3.75 × 10^−3^
	HI	3.52 × 10^−1^	5.51 × 10^−3^	3.75 × 10^−3^	5.10 × 10^−3^	3.85 × 10^−3^

**Table 6:** Alpha diversity levels of microorganisms in monitoring wells.

Monitoring Well	Chao1	Shannon	Simpson
HLJ2	2746	0.0955	3.88
HLJ3	4936	0.0761	4.73
HLJ4	4987	0.0364	5.39
HLJ5	5179	0.042	5.18
HLJ6	3276	0.1047	4.07

## Data Availability

Data is unavailable due to privacy or ethical restrictions.

## Supplementary Materials

The following supporting information can be downloaded at: https://www.mdpi.com/article/10.3390/ijerph20010604/s1, Table S1: Exposure parameters of health risk assessment model; Table S2: Skin permeability coefficient (PC), slope factor (SF) and reference dose (RfD).
