# Supplementary material for: Health Risk Assessments and Microbial Community Analyses of Groundwater from a Heavy Metal-Contaminated Site in Hezhou City, Southwest China
Source: Int J Environ Res Public Health. 2022 Dec 29;20(1):604. doi: 10.3390/ijerph20010604 (PMC9819039; doi:10.3390/ijerph20010604)
Supplement: Supplementary file 1 [file ijerph-20-00604-s001.zip › ijerph-2098848-supplementary.pdf]

Supporting information

Health risk assessments and microbial community analyses of groundwater from a heavy metals-contaminated site in Hezhou City, Southwest China

Mingjie Xu[a], Kuankuan Zhang[b], Yiduo Wang[c], Bin Zhang[a, d, *], Kang Mao[b], Xue jiao Chen[d], Hua Zhang[b,*]

[a] *School of Architecture and Civil Engineering of Xihua University, Chengdu, 610039, China*

[b] *State Key Laboratory of Environmental Geochemistry, Institute of Geochemistry, Chinese Academy of Sciences, Guiyang, 550081, China*

[c] *College of Eco-Environment Engineering, Guizhou Minzu University, Guiyang 550025, China*

[d] *School of food and biotechnology of Xihua University, Chengdu, 610039, China*

Table S1 Exposure parameters of health risk assessment model

Symbol	Parameters	Units	Adult	Child
IR	Ingestion rate	L/d	1.7	1.14
BW	Average body weight	kg	57	23.8
EF	Average exposure frequency	d/a	350	350
ED	Average exposure duration	a	26	6
SA	Exposed Area of Skin	cm^2	18000	6600
CF	Volume conversion factor	L/cm^3	0.001	0.001
ET	Exposure Time	h/d	0.58	1
AT_{nc}	Average time for non-carcinogenic effect	d	2190	2190
AT_{ca}	Average time to carcinogenic effect	d	26280	26280

Table S2 Skin permeability coefficient (PC), slope factor (SF) and reference dose (RfD)

	**PC** (**10^{-3}cm/h**)	**SF_o** (**mg·（kg·d）$^{-1}$**)	**SF_d** (**mg·（kg·d）$^{-1}$**)	**RfD_o** (**mg·（kg·d）$^{-1}$**)	**RfD_d** (**mg·（kg·d）$^{-1}$**)
As	1.8	1.5	3.66	0.0003	0.0001
Cd	2	6.1	0.38	0.0005	0.000005
Cr	2	0.5	20	0.003	0.00006
Pb	0.004			0.0014	0.0014
Mn	0.1			0.046	0.0018
Cu	1			0.04	0.012
Ni	0.1			0.02	0.0054
